# Supplementary material for: MetaRibo-Seq measures translation in microbiomes
Source: Nat Commun. 2020 Jun 29;11:3268. doi: 10.1038/s41467-020-17081-z (PMC7324362; doi:10.1038/s41467-020-17081-z)
Supplement: Supplementary file 10 — Supplementary Data 7 [file 41467_2020_17081_MOESM10_ESM.zip › File2/Confidence_High_Taxonomy/212396_out.krona.html]

Javascript must be enabled to view this page.

members
magnitude
magnitudeUnassigned
count
unassigned
taxon
rank

212396\_out

2

superkingdom
2
2

phylum
1239
2

186801
2
class

2
186802
order

family
2
186803

572511
2
genus

2
1226324
species

SRS077502\_contig\_number\_8284SRS1041091\_contig\_number\_18445
